# Different Clinical Features of Acral Abortive Hemangiomas

**DOI:** 10.1155/2017/2897617

**Published:** 2017-07-12

**Authors:** N. Vega Mata, J. C. López Gutiérrez, B. Vivanco Allende, M. S. Fernández García

**Affiliations:** ^1^Department of Pediatric Surgery, Hospital Universitario Central de Asturias, Oviedo, Spain; ^2^Department of Pediatric Surgery, Hospital La Paz, Madrid, Spain; ^3^Department of Pathology, Hospital Universitario Central de Asturias, Oviedo, Spain

## Abstract

Some infantile hemangiomas called in literature “minimal or arrested growth hemangiomas” or “abortive hemangiomas” are present at birth and have a proliferative component equaling less than 25% of its total surface area. Often, they are mistaken for vascular malformation. We present five patients (three girls and two boys) with abortive hemangiomas diagnosed between January 2010 and December 2015 localized in acral part of the extremities. They were congenital lesions resembling precursor of hemangiomas but did not show proliferation phase. Immunohistochemical Glut-1 was performed in all of them as a way to confirm the abortive hemangioma diagnosis. The most common appearance was a reticulated erythematous patch with multiple fine telangiectasias on the surface. We remark that one of them presented a segmental patch with two different morphologies and evolutions. The proximal part showed pebbled patches of bright-red hemangioma and presented proliferation and the distal part with a reticulated network-like telangiectasia morphology remained unchanged. We detected lower half of the body preference and dorsal region involvement preference without ventral involvement. The ulceration occurred in three patients with two different degrees of severity.

## 1. Introduction

Infantile hemangiomas (IH) are the most common tumour of infancy and childhood, usually absent at birth or present as a premonitory mark (e.g., pink macule, telangiectatic patches, or bruise-like area). The hallmarks of IH are their 3 clearly defined stages: they appear in the first weeks of life and undergo a rapid growth phase, followed by a period of slow involution with possible residual lesions [1, 2]. This typical evolution makes them easy to diagnose clinically in most cases. In addition, IH express an erythrocyte-type glucose transport protein (GLUT-1) in their endothelial cells, which is a highly specific marker of IH [1, 3].

Some IH have a proliferative component equaling less than 25% of its total surface area. They resemble premonitory marks and they have been called in medical literature “minimal or arrested growth hemangioma”(IH-MAG) or “abortive hemangiomas.” Diagnosis of IH-MAGs can be difficult because they can present as a macular infantile hemangioma with a network-like and blotchy appearance and they are often mistaken for vascular malformation [4]. We describe the clinical, histological, and evolution data of five patients IH-MAGs involving the lower extremities showing atypical and characteristic clinical course. The immunohistochemical Glut-1 confirmed the IH diagnosis. Clinical features, complications, and evolution are analyzed.

## 2. Methods

We report five patients seen between January 2010 and December 2015 with IH that did not show postnatal proliferation. All patients did not have family history of similar lesions and the rest of their physical examination was normal. We performed a punch biopsy and immunohistochemistry in all cases. GLUT-1 positive immunostaining was seen and it confirmed that it was IH-MAG.

## 3. Case  1

**Figure 1:**
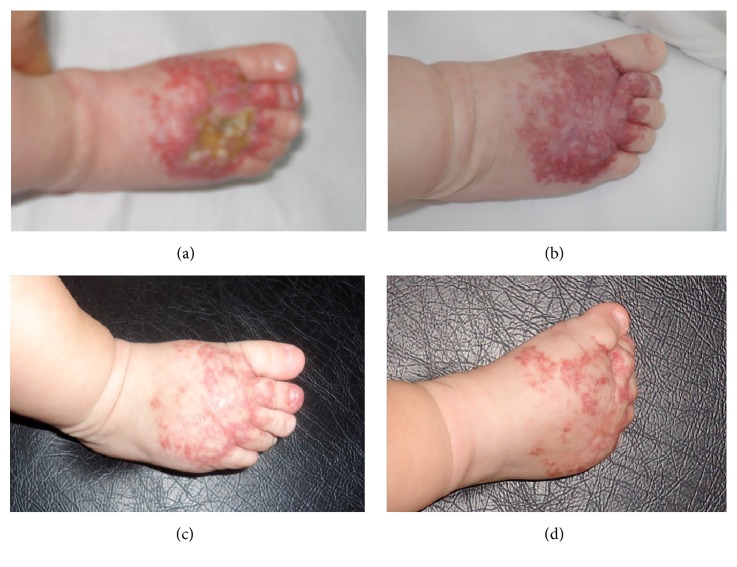
Case  1. (a) Ulceration on an erythematous patch with a network-like and blotchy appearance in the dorsal surface of the foot of a boy of 3 months. (b) Resolution of the ulceration after 1 month with propranolol treatment. Progressive regression of the lesion at 9 months (c) and 11 months (d).

An 11-month-old Caucasian boy presented at birth an erythematous patch with a network-like and blotchy appearance in the dorsal surface of his right foot that extended onto the digits but not to the distal tip (Figure 1). He was born full-term weighing 3240 g, the result of the mother's second pregnancy and an eutocic delivery. The lesion did not proliferate but he was referred to our centre at 3 months of life for a deep ulceration within the erythematous patch. The ulceration did not respond to topical and systemic antibiotic and it produced an important pain. So, a skin biopsy of 5 mm was carried out and the histopathology revealed ectatic vessels in the dermis without lobular pattern. Endothelial cells of these vessels were positive for anti-Glut-1 immunostaining compatible as abortive hemangioma. We initiated treatment with propranolol (3 mg/Kg/day) and the patient presented a good response. The ulcerative lesion healed and the erythematous patch started an involutive evolution without complication.

## 4. Case  2

**Figure 2:**
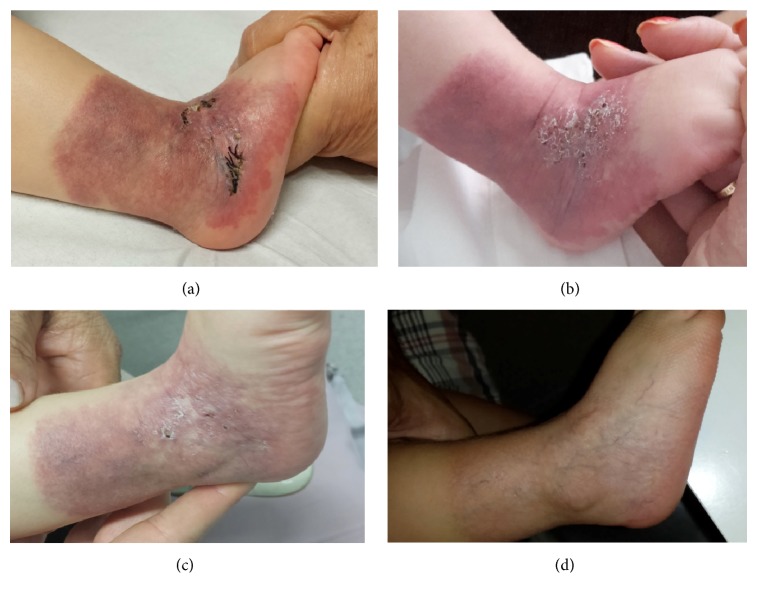
Case  2. (a) A skin biopsy on erythematous scaly plaque with geometric border covering all lateral aspect of her right ankle as a sock-like distribution. (b) Numerous superficial ulcerations of this erythematous scaly plaque. (c) Progressive regression of the lesion and the ulceration with propranolol treatment. (d) Spontaneous involution of the lesion.

A 4-month-old Caucasian baby girl presented at birth an erythematous scaly plaque with geometric border covering all lateral aspect of her right ankle as a sock-like distribution was seen. She was born full-term weighing 3.270 g, the result of the mother's first pregnancy and an eutocic delivery, with no personal or family history of interest. This plaque had bright-red papules, telangiectatic in appearance with a surrounding area darker in colour (Figure 2). The rest of the physical examination was normal. The lesions remained stable and there was no sign of proliferation but over the first week of life the lesion presented numerous superficial ulcerations, resulting in severe pain. A skin biopsy of 5 mm was carried out and the histopathology revealed a hyperkeratosis epidermis and ectatic vessels in the papillary and reticular dermis without lobular pattern. In the reticular dermis, there were plump endothelial cells and ectatic thin-walled vascular spaces. Endothelial cells were positive for anti-GLUT-1 immunostaining. Due to ulceration, we initiated treatment with propranolol (3 mg/Kg/day) and the patient presented a quick involution of hemangioma for four months. After, she had to discontinue the treatment because of an episode of bronchiolitis and the hemangioma continued with a slowly spontaneous involution. In this moment, she is one and a half years old and she still has residual lesions composed of a reticulated patch with a network-like telangiectasia, a darker periphery with anemic areas, and spontaneous involution is present.

## 5. Case  3

**Figure 3:**
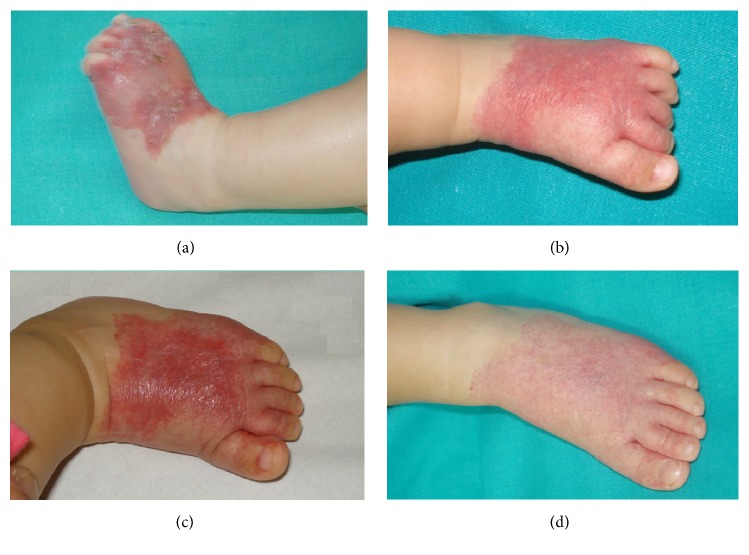
Case  3. (a) An erythematous scaly patch in the dorsal and lateral surface of the left foot extending onto the digits which presented multiple and superficial ulcerations. (b) Response to propranolol after the first month of treatment. (c) Response to propranolol at sixth months of treatment. (d) Spontaneous involution.

A 2-month-old Caucasian girl presented at birth an erythematous scaly patch in the dorsal and lateral surface of the left foot extending onto the digits (Figure 3). She was born full-term weighing 3460 g. The plaque did not present proliferation but, as case 2, over the first week of life it presented numerous superficial ulcerations, resulting in severe pain. A skin biopsy of 5 mm was carried out and the histopathology revealed ectatic vessels in the papillary and superficial reticular dermis without a lobular pattern and the anti-GLUT-1 antibody was positive. We initiated treatment with propranolol (3 mg/Kg/day) for 6 months and the patient presented a good response. The ulcerative lesion scared and it started an involutive evolution. In this moment, she presents a residual lesion composed of a reticulated patch with a network-like telangiectasia and spontaneous involution is present.

## 6. Case  4

**Figure 4:**
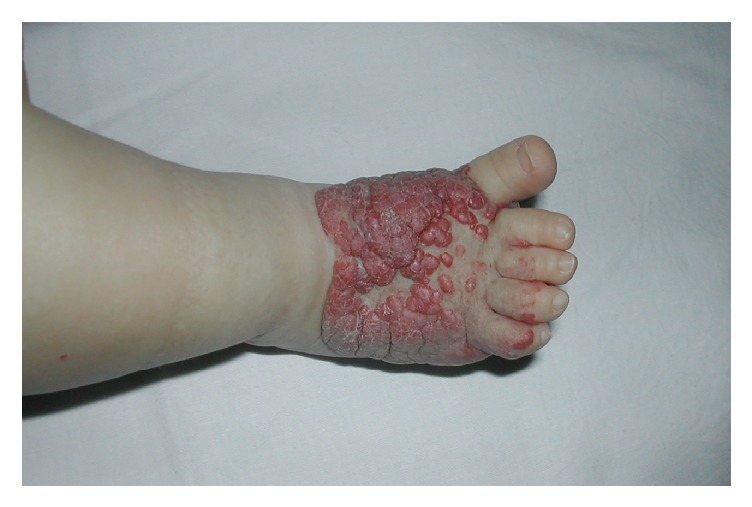
Case  4. Pebbled patches of bright-red hemangioma and the distal part extending onto the digits presenting reticulated network-like telangiectasia morphology.

An 8-month-old Caucasian boy was referred to our centre for a segmental patch with two different morphologies. The proximal part presented pebbled patches of bright-red hemangioma and the distal part extending onto the digits presented a reticulated network-like telangiectasia morphology (Figure 4). He was born in the 40th week of an uncomplicated pregnancy. The lesion had been present from birth, the proximal part presented proliferation, but the distal part remained unchanged. A proximal and a distal 4 mm punch biopsy were performed and both revealed ectatic vessels in the papillary and superficial reticular dermis with lobular pattern. The endothelial cells of both superficial and deep vessels were positive for anti-GLUT-1 immunostaining. The lesion did not present ulceration. We initiated treatment with propranolol (3 mg/Kg/day) and the hemangioma started a progressive involution but we did not have pictures to show the involution of the lesion because the patient missed the follow-up.

## 7. Case  5

**Figure 5:**
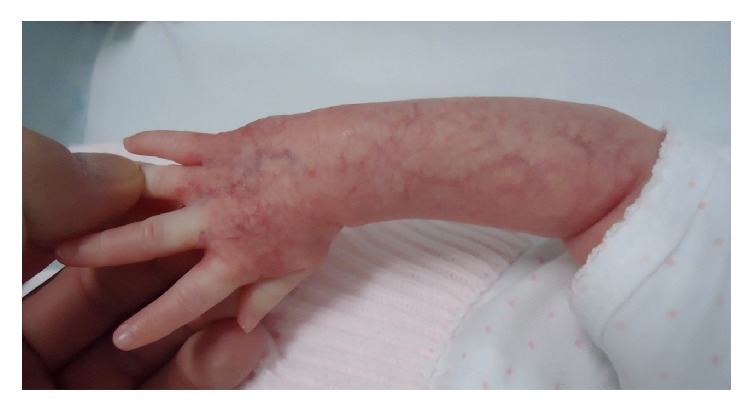
Case  5. An erythematous patch with prominent surface telangiectases on the dorsal arm of a 3-month girl.

A 3-month-old Caucasian girl presented at birth an erythematous patch with prominent surface telangiectases on her dorsal arm (Figure 5). She was born at 39 weeks of gestation after an uncomplicated pregnancy. The telangiectatic macule had remained unchanged but due to family anxiety a biopsy was performed. Histology showed ectatic vessels in the papillary dermis without a lobular pattern and GLUT-1-positive vessels were seen. Then, a reticular abortive hemangioma was diagnosed. The patient was asymptomatic so we decided to maintain an expectant management and wait for a slowly spontaneous involution.

## 8. Discussion

The characteristic clinical features at birth of IH and of congenital haemangioma are well established. HI are usually absent at birth or present in one-third as a premonitory mark in the form of telangiectatic papules, pale pinkish maculae, or mottled vascular stain. Its hallmark is its rapid postnatal growth beginning 2 weeks after birth and followed by slow involution [2]. However, congenital hemangiomas, both major subtypes RICH and NICH, are fully grown at birth. The regression in RICH usually starts a few days to a few weeks after birth, and complete resolution usually occurs by 14 months. The NICH do not resolve spontaneously and the tumour grows proportionately with the child [5].

In our five cases, the lesions were present at birth and showed neither the typical morphology present in a RICH nor NICH. Then as the clinical presentation was not typical for either a congenital haemangioma or an IH, vascular malformation was considered in the differential diagnosis. A biopsy for histology study and Glut-1 antibody staining was performed in all cases. Glut-1-positive immunostaining was seen, confirming that it was true IH and not congenital hemangioma (RICH or NICH) or vascular malformation [5]. In particular, those lesions present at birth that do not exhibit a postnatal growth were IH-MAG a subtype of IH. GLUT-1 is an established specific marker for IH and is useful in terms of distinguishing it from other paediatric vascular tumours or vascular malformations. It would have been very difficult to differentiate them from vascular malformation without the aid of GLUT-1 staining [3, 4, 6].

We present these five cases of IH-MAGs with three different morphologies localized on acral part of the extremities. The most common appearance was as reticulated erythematous patches with multiple fine telangiectasias on the surface as in cases 2, 3, and 5. The first patient had an erythematous patch with a network-like and blotchy appearance in the dorsal surface of his right foot that extended onto the digits. The fourth case presented a segmental patch with two different morphologies. The proximal part exhibiting pebbled patches of bright-red hemangioma and the distal part extending onto the digits had a reticulated network-like telangiectasia morphology. This last morphology had not been described before and in our experience we have not seen the other two morphologies described in IH-MAGs: grouped telangiectasias overlying normal-appearing skin or bluish plaques with normal or a pale peripheral halo [1].

We reviewed the literature and we detected that IH-MAG frequently involved the lower half of the body (57% [1], 100% [5], 41% [7], 64% [8], and 68% [9]), and a high percentage of them affected acral part. In addition, these acral IH were more likely to have a predominantly reticular morphology. Suh and Frieden have reported that IH-MAGs were 26 times more likely than IH to appear on the lower half of the body compared with the upper half of the body [10]. Our experience supports this hypothesis as 4 of our 5 cases were located on the foot. We emphasize that all our patients had the dorsal region involved. If we review the literature we realize that hemangiomas in the ventral part of acral extremities present neither arrested growth nor ulceration. Perhaps, the different characteristics of the skin in the dorsal or ventral surface of acral extremities change the evolution of these lesions. Another important characteristic of this subtype of IH was its predominance in girls and Caucasian race. We also support this predilection for the female sex and Caucasian race because three of our patients were females and all of them were Caucasians [10].

The higher incidence of hemangiomas in Caucasian population, females, and premature infants could be related to higher level of renin found in these groups of populations [6]. In fact, some authors have demonstrated the involvement of the renin-angiotensin system (RAS) in the biology of IH. This is based on the expression of angiotensin-converting enzyme and angiotensin II receptor 2 on the endothelium of proliferating IH-derived blast cell proliferation [7]. Certainly, RAS play an important role in the biology of IH and potentially to IH-MAG but more research is still needed in order to elucidate the exact role of RAD in IH-MAG.

A subtype of IM-MAG with a predominantly reticular pattern was described by Mulliken as reticular hemangioma (RIH-MAG). It is characterized by severe complications including intractable ulceration, tissue, and bone destruction. Additionally it can be associated with structural anomalies [8, 11]. Ulceration was a common finding in our group. It occurred in two patients with reticular morphology and in one patient without this reticular pattern [9, 12]. We detected two different degrees of involvement: ulcerations in two patients (cases 2 and 3) were numerous and superficial but in another patient (case 1) the ulceration was focal, deep, and difficult to heal.

The presence of ulceration in an IH-MAG suggests that proliferation alone may not be the sole reason and that other mechanisms such as hypoxia or local environment may have a role.

The natural history of IH-MAG is regression as other forms of infantile hemangioma. We detected that the involution phase is slower than usual and begins by about 1 year of age. Some of them do not require any treatment other than observation. The need for treatment in acral IH-MAG comes from ulceration showing a good response to propranolol as normal IH. They can leave residual lesions composed of a slightly puffy skin and telangiectasias.

## 9. Conclusion

We present a subtype of infantile haemangioma with similar clinical course: present at birth, located on the dorsum of feet, and showing minimal and arrested growth and developing characteristic ulceration. We highlight the importance of performing immunohistochemical Glut-1 examination in order to confirm diagnosis when clinical features are atypical. IH-MAGs were more likely than IH to appear in girls, on the lower half of the body and on dorsal surface. Acral abortive hemangiomas were more likely to have a predominantly reticular morphology. Ulceration is a frequent complication of acral abortive hemangiomas.
